# Real-World and Regulatory Perspectives of Artificial Intelligence in Cardiovascular Imaging

**DOI:** 10.3389/fcvm.2022.890809

**Published:** 2022-07-22

**Authors:** Ernst Wellnhofer

**Affiliations:** Institute of Computer-Assisted Cardiovascular Medicine, Charité University Medicine Berlin, Berlin, Germany

**Keywords:** machine learning (ML), regulation, innovation, software as a medical device (SaMD), safety and risk, total product life cycle (TPLC)

## Abstract

Recent progress in digital health data recording, advances in computing power, and methodological approaches that extract information from data as artificial intelligence are expected to have a disruptive impact on technology in medicine. One of the potential benefits is the ability to extract new and essential insights from the vast amount of data generated during health care delivery every day. Cardiovascular imaging is boosted by new intelligent automatic methods to manage, process, segment, and analyze petabytes of image data exceeding historical manual capacities. Algorithms that learn from data raise new challenges for regulatory bodies. Partially autonomous behavior and adaptive modifications and a lack of transparency in deriving evidence from complex data pose considerable problems. Controlling new technologies requires new controlling techniques and ongoing regulatory research. All stakeholders must participate in the quest to find a fair balance between innovation and regulation. The regulatory approach to artificial intelligence must be risk-based and resilient. A focus on unknown emerging risks demands continuous surveillance and clinical evaluation during the total product life cycle. Since learning algorithms are data-driven, high-quality data is fundamental for good machine learning practice. Mining, processing, validation, governance, and data control must account for bias, error, inappropriate use, drifts, and shifts, particularly in real-world data. Regulators worldwide are tackling twenty-first century challenges raised by “learning” medical devices. Ethical concerns and regulatory approaches are presented. The paper concludes with a discussion on the future of responsible artificial intelligence.

## Introduction

Artificial intelligence (AI), machine learning (ML), and deep learning (DL) are often used interchangeably. This pars pro toto usage of terms is rooted in the growing importance of DL in ML and ML in AI. DL is a subset of ML, and the latter is a subset of AI. AI is used as a term for artificial systems that perceive and process input data and achieve specific goals. In changing data ecosystems and application contexts, adaptive learning or even problem-solving may be essential functions of such systems (1, 2). The popular concept of characterizing AI as an emulation of human cognitive performance or “strong AI” may be a general ethical concern in the future but does not apply to current cardiovascular imaging applications.

ML/DL maps data patterns on output without intermediate hypothesis modeling. A strength of this approach is the ability to discover an unknown pattern in the data and derive models without requiring *a priori* assumptions about frequently poorly understood underlying features. The algorithms are trained and tuned on a sufficiently large set of representative data and evaluated on a separate hold-out test set of data. Training is iterative and guided by a cost function. The cost function may be based on the difference between (partially) known (labeled) ground truth and algorithmic estimate called supervised learning (semi-supervised learning), on intrinsic data patterns termed unsupervised learning, or on incentives called reinforcement learning. Competing cost functions may be employed as adversarial learning, e.g., for recognizing fake images. A comprehensive survey on ML methods cannot be integrated into this paper because the field is an evolving science with many ramifications ranging from statistical learning to neuromorphic computing (3). A concise overview of ML methods used in cardiovascular imaging may be found in recent papers (4, 5). “A deep-learning architecture is a multilayer stack of simple modules, all (or most) of which are subject to learning, and many of which compute non-linear input-output mappings” (6). Supervised or semi-supervised DL was successfully used in image segmentation and classification. Convolutional neural nets (CNN) are a specialized kind of neural network particularly suited for processing data with a known grid-like topology (6). Neural nets are hierarchically stacked layers of modules that are partially or fully connected. The first layer is the input vector, e.g., a vectorized pixel matrix of the image to be analyzed. The hidden layers extract features on different scales. The last layer is the output vector representing the result of classification or segmentation. The input of a module is the sum of weighted outputs of the modules of previous layers activated by a non-linear function. Convolutional layers may be seen as additional self-learning filters critical in imaging applications. Spatial pooling reduces spatial resolution and controls the size of the layers. Residual connections skip layers and thereby stabilize very deep nets with more than 150 layers. An appropriate cost function guides learning optimal weights for connections and filters. Details and research on more technical issues, e.g., optimization of learning and avoiding overfitting, may be accessed as HTML MIT Press book.^1^ We refer to a recent paper for more detail on cardiovascular imaging (7). DL by CNN requires enormous computing resources. The calculus is simple algebra, however, and can be massively parallelized. Thus, advances in computing hardware, particularly graphic processing units and parallel computing resources, enabled these approaches. Big data are the fuel of DL. The ImageNet challenge and other annual competitions^2^ sped up the evolution of DL. A worldwide open-source community further promoted the rise of DL. User-friendly software environments, e.g., Caffe,^3^ Tensorflow,^4^ Theano,^5^ PyTorch,^6^ Lasagne,^7^ and Keras^8^ enable scientists worldwide to explore DL.

As opposed to large amounts of available images, e.g., on ImageNet, moderate or small sets of cardiovascular images are often confined to local picture archiving systems (PACS) due to data protection laws and privacy concerns. Attempts to pool medical images struggle with interoperability issues and legal constraints. There are different approaches to solve the problem of limited health data comprising data augmentation, construction of representative artificial images, deidentification of data sets, various models of federated learning, and secure pooling using blockchain ledger technologies. The problem of small data sets may also be approached by tweaking the nets, pre-training on non-medical images, and fine-tuning on health images. Last but not least medical PACS systems are evolving into big data repositories in our digital ecosystem. According to Badano et al., artificial intelligence and cardiovascular imaging are a win-win combination (8).

AI in cardiovascular imaging is a medical device. Patients and physicians expect approved or certified medical devices to be safe and effective^9^ (9). They also desire to profit from innovations in medicine due to novel technologies. However, assessing safety and efficacy consumes time and may result in a roadblock to fast and agile innovation. Competent authorities must manage to provide responsible regulation without quenching innovation. This is a growing challenge in software as a medical device (SaMD) or part of a medical device. Software is increasingly crucial for efficacy in novel state-of-the-art medical devices. Yet, the software may be complex and even change by ‘‘learning’’ [machine learning medical devices (MLMD)^10^ ]. As it is more difficult to aim at a moving target than a fixed one, regulating learning frequently changing or even autonomous medical devices is more demanding than regulating medical devices in the past that were rarely ever changed. Whereas, external supporting evidence was manually gathered data in the past, we currently wrangle with a flood of digital data. This big data comprises archived data collected with other medical purposes, e.g., medical images in PACS systems. MLMD thrives on the ingestion of such big data. Consequently, regulation has to deal with a garbage-in-garbage-out risk of data sources tapped by MLMD devices. Bias, errors, drifts, and shifts in data may entail unanticipated and unintended output. Suppose data is mapped on the output by complex non-linear and implicit feature extraction, e.g., in neural networks. In that case, a check against the hypothesis as in classical science is lacking. Thus, judging the validity of the outcome may be challenging, and explainable AI becomes an issue. New technologies need new ways of regulation.

The remainder is a sketch of current cardiovascular MLMD imaging, ethical concerns, current state-of-art, and emerging regulatory changes. In the first subsection, the use of MLMD in cardiovascular imaging is briefly exposed. Ethical and legal concerns and specific risks of MLMD are discussed in the second subsection. The third subsection presents the basics of SaMD and MLMD regulation, relevant standards, some existing regulatory environments and a comparison of the EU and the USA with examples that entail a reflection on autonomy. The discussion starts with a short summary and addresses the future of MLMD cardiovascular imaging, digital data, and evolving regulation.

## Real-World and Regulatory Perspectives

### Current State of Artificial Intelligence and Machine Learning in Cardiovascular Imaging

The scope of this section is to describe the broad range of actual and potential applications and provide suitable references for readers that are not familiar with all applications “of AI in CV Medicine.” A detailed review of applications of MLMD in cardiovascular imaging is covered by many recent papers (10–17).

There is a wide range of applications comprising image acquisition (18, 19), pre-processing, segmentation, automated measurements, quality control, image retrieval, matching real-time acquired images with previous images (20), fully automated quantitative assessment (21), and even diagnostic guidance^11^ and diagnosis. MLMD may enable personalized treatment based on cardiovascular imaging phenotypes and prognostic stratification. Essential issues are automatic screening of unread studies for potential emergency findings requiring urgent reporting and emergency treatment. Meanwhile, medical devices that provide fully automated quantitative assessments of images are commercially available for different modalities as echocardiography (22, 23), cardiac magnetic resonance imaging (CMR), computed tomography (CCT) (24, 25), nuclear cardiac imaging (25, 26) and multimodality imaging (17, 25).

Radiomics is a recent sophisticated feature exploiting approach initially conceived as an image biomarker approach in personalized cancer treatment that is a rising star in cardiovascular imaging and precision medicine. Radiomics in computed tomography comprises intensity-based, texture-based, shape-based, model-based, and transform-based metrics (27, 28). The Radiological Society of North America (RSNA) initiated a Quantitative Imaging Biomarkers Alliance (QIBA),^12^ and the European Society of Radiology (ESR) merged the activities of the former ESR Subcommittee on Imaging Biomarkers, the ESR Working Group on Personalized Medicine, and ESR-EORTC Working Group into the European Imaging Biomarkers Alliance (EIBALL).^13^ Methods in radiomics are still a research area, however. Radiomic features are sensitive to image acquisition settings, reconstruction algorithms, and image processing. Strict adherence to standardized image acquisition and reconstruction protocols, including standardization, harmonization, and feature reduction techniques, is recommended. Major pitfalls are class imbalance, overfitting, and sometimes underfitting. Nuclear medicine is expected to profit from this research tool in clinical decision-making and discovering novel molecular pathways (28).

As the clinical evaluation of MLMD entails clinical investigations, appropriate amendments to reporting guidelines should be accounted for Liu et. (29), Cruz et. (30). Developing appropriate study protocol extensions for MLMD is ongoing (31, 32).

Within the last 5 years, more than 1,814 full texts on MLMD in cardiovascular imaging were published. A literature search algorithm allowing regular updates for cardiovascular care is provided by Quer et al. (33).^14^

### Specific Risks by Artificial Intelligence and Machine Learning-Ethical and Legal Concerns

The promises of MLMD come with new risks due to unexpected behavior and specific weaknesses of the method. Input attacks, e.g., are surreptitious cyber-attacks by manipulating input data. Hidden bias is deeply rooted in data. Limitations and flaws of human judgment, prejudices, and fashions leave traces in data. Moreover, even corrupted data may betray sensitive information like race, eluding awareness of clinical experts (34). And last not least, spurious data correlations may lead to mismatch (35), famous in MLMD as “Clever Hans” (36). Model predictions must be evaluated with confidence limits of uncertainty in mind (37). Other MLMD-specific problems are potentially unpredictable behavior, lack of transparency and under-specification, and problems during deployment and use of AI systems. Under-specification is common in modern machine learning pipelines and causes real-world performance impairments and failure of generalization. Sensitivity analyses, stress- tests regarding requirements, and the development of application-specific regularization schemes may help to control this issue (38).

Health, and cardiac imaging, in particular, is a sensitive and safety-critical domain. MLMD should be accurate, robust, trustworthy, transparent, explainable, understandable, and resilient. There may be tradeoffs between goals that depend on stakeholders and context (39).

The Wellcome report on ‘‘ethical, social, and political challenges of artificial intelligence in health’’^15^ addresses several scenarios of medical socio-technical application contexts including MLMD-agents such as process optimization, preclinical research, clinical pathways, and patient-facing and population-level applications. The report identified the most critical challenges across use-cases: There was concern about the impact of AI on human relationships, data management, governance, and existing health inequalities. Further questions regarded issues around transparency and explainability of algorithms in health settings, software-generated as opposed to human decisions, patients’ and public expectations from AI and related technology, best practices of regulation, eventually forbidden use of new information, the trustworthiness of algorithms, and last not least problems arising from the private-public collaboration. In conclusion, there are three major areas of concern, consent, fairness, and rights. If there is some autonomy in AI’s decision that might not be fully understood, what means consent to use this service? Distributive fairness is conventionally governed by three principles (responsibilities, capabilities, and needs). Yet, the application of these fuzzy concepts strongly depends on interpretation. Moreover, MLMD is a rapidly changing technology. New approaches to specify and warrant fairness in these novel socio-technical contexts should be considered. An issue regarding rights spins around the point of whether there is a right to human delivery of healthcare. In the European Union, citizens have the right not to be subject to automatic decisions solely without any human intervention and to get an explanation of a decision’s logic.^16^

At the population level, there seem to be three main expectations: the final responsibility of human physicians for diagnosis and treatment, explainable MLMD decision-support, and testing of the system for discrimination (40). The European Commission set up a High-Level Expert Group on Artificial Intelligence^17^ that elaborated ethics guidelines for trustworthy AI (41) based on a human-centric approach. AI is not an end in itself but a tool that has to serve people with the ultimate aim of increasing human wellbeing. Trustworthy AI should be lawful, ethical, and robust. The guideline focuses on ethics and robustness. AI should be developed, deployed, and used based on four principles: respect for human autonomy, harm prevention, fairness, and explicability. Particular attention should be paid to potential tensions between these principles and the impact on vulnerable groups, e.g., children or disabled persons. The vision of benefits should not impair the scrutiny of pending risks and adequate mitigation. In chapter II, guidance is detailed. AI should be developed, deployed, and used meeting ‘‘seven key requirements for Trustworthy AI: (1) human agency and oversight, (2) technical robustness and safety, (3) privacy and data governance, (4) transparency, (5) diversity, non-discrimination and fairness, (6) environmental and societal wellbeing, and (7) accountability.’’ Technical and non-technical methods should achieve the implementation of these requirements. Technical approaches include specific architecture, ethics, and the rule of law by design, explanation tools, rigorous testing and validation that should include adversarial/penetration testing, and quality of service indicators. Non-technical methods comprise regulation, codes of conduct, standardization, certification, governance, education, ethical mindset, stakeholder participation, diversity, and inclusive design teams. The guideline recommends fostering research, innovation, dissemination of information, and education on ethics in AI. Communication with stakeholders regarding capabilities, limitations, and expectations of AI applications should be clear, proactive, and transparent. AI systems should be traceable and auditable. All stakeholders^18^ should be involved throughout the total lifecycle of the product. Fundamental tensions between different principles and requirements need continuous attention. Chapter III provides a preliminary checklist for assessing trustworthy AI for tailoring and continuous improvement. The current final version of the ‘‘Assessment List for Trustworthy Artificial Intelligence (ALTAI) for self-assessment’’^19^ is available as PDF or web-based tool.

The term meaningful human (MHC) control originated from the debate on autonomous weapons and the danger of accountability gaps. What is MHC? In real-world environments comprising humans and technology, particularly in the increasingly complex healthcare environment, MLMD is embedded in a human agent team (HAT) (42). A HAT is a pit crew where human generalists, specialists, and technical agents collaborate in specific contexts on common tasks. Assessments of MHC comprises “experienced MHC” and behavioral alignment with ethical guidelines and moral values. In a HAT, an accurate mental model of the MLMD agents is indispensable (42). A team’s mental model is a shared framework that provides unambiguous roles of technical and human agents, mutual trust, and exchange of information. It comprises a team model, a team interaction model, an equipment model, and a task model. The necessary explanations of technical agents that support human-agent collaboration in such a context are provided by eXplainable Artificial Intelligence (XAI). However, explanations may depend on teams, contexts, and tasks (43, 44). Thus, an actionable implementation of XAI can be based on a mental models approach (44). Explainability^20^ is a fuzzy concept that is often conflated with interpretability, causability, or understandability. In technical philosophy, an explanation is a mapping of something to be explained on an explanation. Interpretation may transform a poorly understood explanation into a more understandable one. Approaches to interpretation may be total or partial, global or local, approximative or isomorphic (45). Explainability in XAI technically identifies features and mechanisms that decide the performance and the outcome of an MLMD system. Whereas, interpretability may be understood as a broader concept. Thus a recent review titled: “Explainable AI: A Review of Machine Learning Interpretability Methods” (46). This thorough review of the art of interpretation provides six comprehensive tables of published methods based on a taxonomy of methods and an appendix with repository links. The term causability addresses the causal-mechanistic understandability or evidence of an explanation for experts in a specific context. Understanding comprises perceiving and reasoning based on a model of reality. Levels of causability are association, investigational intervention, and counterfactual reasoning. Causability should be integrated analogous to usability^21^ as an essential requirement in MLMD applications. The quality of explanations must be developed as a critical safety measure in HATs comprising MLMD (47).^22^

Safe and effective implementation of ethical considerations into devices and their use may be achieved by embedding ethical values in the total product life-cycle. Targets in development are intended use, requirements, design, and architecture (48, 49). Various approaches comprise integrating ethics in structures and processes in development (50), embedding micro-ethics and virtues into data science (51) and enforcing ethical safety and trust by improving regulation (52). The rest of the paper will be confined to depicting existing regulatory frameworks and recommendations for future regulation.

### Existing Regulatory Frameworks

The scope of regulation is to make sure that only effective and safe medical devices are on the market. Thus, patients and providers may rely on a competent authority or regulating body’s approval or certification of a device.

Effectiveness is evaluated by validation of the intended use of the device. Safety, including safe use, is the outcome of risk management. Regulatory bodies continuously react to manifest serious incidents and may proactively adapt regulation to evolving risks due to novel technologies and anticipated risks. There may be some tension, however, between responsible regulation and innovation.

The International Medical Device Regulators Forum (IMRDF)^23^ was established in October 2011 by regulatory bodies worldwide and focuses on approaches for rapidly evolving technologies in medical devices and ‘‘on developing a total product lifecycle approach (TPLC) to regulating medical devices while enabling timely access to safe access and effective devices for the patients.’’ A harmonized approach to the management of MLMD is a strategic priority.^24^

The current regulatory policy will be presented by first describing common principles, then pertinent standards reflecting the technical state-of-the-art, and finally special issues in different legislations.

#### General Features of Medical Device Regulation

There are some common features in most regulations for medical devices worldwide:

1. The rigor of regulation is based on the hazard and potential severity of harm. The Global Harmonization Task Force (GHTF)^25^ 2006 published a risk classification approach for medical devices comprising four classes,^26^ adopted in an adapted version in most legislations. The classification is based on the intended use. Low-risk devices may be exempt from regulation. High-risk devices generally need proof of quality, scrutiny and oversight, and evaluation by clinical trials. Classes B (low-moderate risk class II a in EU) and C (moderate-high risk class II b in EU) are combined as an intermediate risk class in the United States.
2. Software, which drives a device or influences the use of a device, falls within the same class as the device.
3. Stand-alone software with an intended use supporting a medical purpose is regulated as Software as a Medical Device (SaMD) and considered or explicitly defined as active^27^ device.
4. Many countries aligned their SaMD regulatory approaches with guidance from the IMRDF.^28^ The guideline entitled ‘‘‘‘Software as a Medical Device’’: Possible Framework for Risk Categorization and Corresponding Considerations’’^29^ classifies risk based on the
  a. Significance of the information provided by the SaMD to the healthcare decision, and
  b. State of the healthcare situation or condition (see Table 1).

**TABLE 1:** Internationally recognized SaMD risk classification according to IMDRF.

State of healthcare	Significance of information provided by SaMD to healthcare decision		
	
	Treat or diagnose	Drive clinical management	Inform clinical management
Critical	IV	III	II
Serious	III	II	I
Non-serious	II	I	I

The use of an MLMD application in a safe context may support a lower risk classification!

5. If there exists a substantially equivalent device (EU) or predicate device (US) demonstrating comparable safety and performance provides access to the market.
6. The product quality of complex medical devices as software and MLMD devices in particular is challenging to ascertain. Therefore, the quality of organizations, structure, and processes in development, deployment, and application is audited adjunctive to rigorous testing.
7. Conflict of interest is an issue in regulatory oversight worldwide. In the United States, a government agency, the Food and Drug Administration (FDA), is in charge of auditing manufacturers. In the EU, private accredited organizations, called notified bodies (NBs), are auditing manufacturers placing devices with higher risk classes than I on the market. To avoid conflict of interest, regulations on accreditation and surveillance of NBs have been increased (see details chapter IV MDR^30^). NBs must not provide consulting services to their clients but may respond to specific questions. The FDA offers the Q-submission service^31^ and the 510(k) Third Party Review Program.^32^
8. Regulation is continuously evolving by amending existing and creating new guidance. New technologies such as MLMD may be addressed by horizontal legislation as intended in the EU by the Artificial Intelligence Act^33^ or vertical integration as proposed by the FDA in the ‘‘Proposed Regulatory Framework for Modifications to Machine Learning Medical Devices (MLMD)-Based Software as a Medical Device.^34^
9. Worldwide continuously learning (and thereby changing) MLMD may not be cleared as medical devices currently!
10. At the moment additional regulatory requirements for MLMD solutions comprise competent staffing, disclosure of data and data management, training and validation protocol with performance metrics and validation results. Specific cybersecurity threats and adversarial attacks in MLMD have to be addressed in the risk-management. Software libraries used for training and testing that are not part of the final device must be validated according to general quality management computer validation requirements (ISO13485). Library code integrated into the final MLMD as a ‘‘predict function’’ has to be validated as software of unknown provenience (IEC 62304) or software of the shelf.^35^ Testing may be performed by test oracles in specialized environments as pytest.^36^ Test coverage is an issue and object of research in complex deep neural nets. Combinatorial testing as efficient black-box approach is recommended. The National Institute of Standards and Technology (NIST) in the United States maintains a Computer Security Resource Center that provides combinatorial testing facilities.^37^ The machine learning community supports tasks, benchmarks, and state-of-the-art methods.^38^
11. As MLMD depends on data, data legislation, data protection and data governance intersect with medical device regulation in this field (53). the Health Insurance Portability and Accountability Act (HIPAA) in the United States.^39^
12. The legal framework of regulation includes procedural and technical requirements that need to be specified in more detail and continuously adapted to state-of-the-art technology. This task is performed by regional and international organizations for standardization (European Committee for Standardization (CEN), European Committee for Electrotechnical Standardization (CENELEC),^40^ American National Standards Institute (ANSI),^41^ National Institute of Standards and Technology (NIST),^42^ British Standards Institution (BSI.),^43^ International Organization for Standardization (ISO),^44^ Institute of Electrical and Electronics Engineers (IEEE),^45^ …).

#### Pertinent Standards

Reference to standards is part of regulatory convergence. MLMD are either stand-alone applications (software as medical device SaMD) or embedded software as part of a medical device. Development of SaMD or embedded software in a medical device compliant with regulation is supported by international standards:

1. On the organizational level
  (a) ISO 13485 Medical devices--Quality management systems-Requirements for regulatory purposes^46^ and
  (b) ISO/IEC 27001^47^ Information technology—Security techniques—Information security management systems—Requirements
2. On the product level
  (a) (IEC 82304-1 Health software--Part 1: General requirements for product safety^48^ for stand-alone software^49^) and
  (b) IEC 60601-1 Medical Electrical Equipment---General requirements for basic safety and essential performance--Collateral Standard: Requirements for medical electrical equipment and medical electrical systems used in the emergency medical services environment^50^ for embedded software
  (c) ISO 14971 Medical devices--Application of risk management to medical devices.^51^
  (d) IEC 62366-1 Medical devices--Application of usability engineering to medical devices.^52^
  (e) ISO 14155 Clinical investigation of medical devices for human subjects--Good clinical practice.^53^
3. On the software development level
  (a) IEC 62304 Medical device software--Software life cycle processes.^54^
  (b) IEC 81001-5-1 Health software and health IT systems safety, effectiveness and security--Part 5-1: Security--Activities in the product life cycle.^55^

There is a rapidly evolving landscape of standards for MLMD. The following is a selection:

1. The ITU/WHO Focus Group on artificial intelligence for health (FG-AI4H) cooperates with the World Health Organization (WHO) to establish standards to assess AI applications in the health sector.^56^ FG-AI4H develops guideline documents on ethical and regulatory considerations (best practices specification), specifications regarding requirements, software lifecycle, data, AI training best practices, evaluation, and scale-up/adoption,^57^ and provides recommendations and tools for auditing and quality controls (37).
2. The joint recommendations regarding machine learning in medical devices of the British Standards Institution (BSI) and the Association for the Advancement of Medical Instrumentation (AAMI) supplement existing regulatory requirements for software as a medical device.^58^
3. The standard ISO/IEC TR 24028 Information technology--Artificial intelligence--Overview of trustworthiness in artificial intelligence^59^ has a scope on trustworthy AI. Requirements to manage AI-specific security, safety, and privacy risks and general requirements for management of weaknesses, threats, and other challenges are proposed based on an analysis of other standards, particularly general risk-management (ISO 31000) and the ISO/IEC 250xx standards series. A strategy is proposed to control bias, manage cybersecurity risks, and provide maximum reliability, resilience, and robustness. Further issues are malfunctions of hardware, improvement of functional security, optimal testing for verification, and appropriate assessment in validation.
4. The Institute of Electrical and Electronics Engineers (IEEE) Global Initiative on Ethics of Autonomous and Intelligent Systems provides the P7000 standards series concerned with ethically aligned design.^60^

Regulation is modified according to legislation. There may be specific additional requirements, amendments, or cross-references. The FDA maintains a list of recognized standards.^61^ In Europe, the commission may require European standardization organizations such as CEN or CENELEC to harmonize an international standard. A European foreword and Z-annexes containing cross-references to MDR are added. Special consultants^62^ check legal conformity. Publication of the harmonized standard in the official journal of the EU confers a legal link to regulation. Conformity with the regulation is presumed if accordance with the standard is demonstrated.^63^ Thus, though even harmonized standards remain voluntary, demonstration of conformity may profit from compliance with harmonized standards. The organizational standard, ‘‘Quality management systems-Requirements for regulatory purposes (ISO 13485)’’, has been harmonized under MDR.^64^ This standard is also widely recognized in other legislations, and a voluntary certification may be advised.

#### Regulation in Europe

MLMD in the EU is regulated as medical device software (MDSW) or software embedded in a medical device by the Medical Device Regulation (MDR).^65^ As far as MLMD applications use data, the General Data Protection Regulation (GDPR)^66^ must be accounted for. The European Commission is currently striving to shape Europe’s digital future. A reassessment is planned in 5 years.^67^

The planned Artificial Intelligence Act^68^ is designed as horizontal legislation. There is an intersection with the vertical MDR, though.^69^ Mandatory requirements for high-risk AI applications are intended. Due to the new classification rules 11 (Medical device software) and 22 (closed-loop systems) in Annex VIII, MDR^70^ medical devices based on or integrating MLMD are likely to be classified as II a or even higher. There is some guidance^71^ on discretion referring to IMDRF policy.^72^ Thus, the use of the MLMD application in a ‘‘safe’’ context may support a lower risk classification. Yet many MLMD devices may end up as high-risk devices that will be covered by mandatory regulation. An impact analysis of the proposed Artificial Intelligence Act with an annex including a stakeholder consultation is published on the EU website.^73^

The CE-mark confirms conformity to the general safety and performance requirements exposed in Annex I MDR and thereby provides access to the European market. As previously discussed, software and MLMD devices may belong to higher risk classes due to the new rules for classification in MDR, Appendix VIII. This implies that a Notified Body (NB) under MDR^74^ has to audit the manufacturer and assess the technical file, including the clinical evaluation report (CEAR). There will be a European Database for Devices (EUDAMED) to ensure the traceability of devices and the transparency of evidence.^75^ Postmarket surveillance and clinical postmarket follow-up (PMCF) are enforced as an early warning system. The MDCG 2019-16 Rev.1^76^ is guidance on cybersecurity for medical devices that addresses the requirements demanded by the MDR.

There are no specific, legally binding requirements for MLMD in Europe at the moment, but notified bodies will check additional requirements according to ‘‘General Features of Medical Device Regulation’’ section 10. Their questions are reflected by the AI-guideline of the Johner Institute.^77^ As dynamically learning and changing AI will not be certified currently applications have to be locked for certification. Outputs that have an impact on diagnosis or treatment should be “explainable.” Otherwise, a conflict may arise with Art 22 and 35 of GDPR.

The NB may consult an expert panel (Clinical Evaluation Consultation Procedure) in high-risk devices. The European Society of Cardiology has established a regulatory affairs committee (54). CORE-MD is a project of stakeholders that seeks to improve research and evidence for high-risk medical devices in cardiovascular, diabetic, and orthopedic medicine by tapping real-world data, establishing registries, and advanced methodology in statistics and trial design. Responsible innovation comprises the evaluation of AI and stand-alone software as a particular focus (55).

#### Regulation in the United States

In the United States, medical devices are regulated by the CFR--Code of Federal Regulations Title 21 Part 800-1299.^78^ The Food and Drug Administration (FDA)’s Center for Devices and Radiological Health (CDRH) is the competent authority in charge of regulation. Regulation is based on risk class. ‘‘Most Class I devices are exempt from Premarket Notification 510(k); most Class II devices require Premarket Notification 510(k), and most Class III devices require Premarket Approval.’’^79^ The FDA provides tailored access to the market by exemptions for investigational devices (IDE) and humanitarian devices (HDE), evaluation of automatic class III designations (*De Novo* Requests), Clinical Laboratory Improvement Amendments, and Breakthrough Devices.^80^

The Q-Submission Program^81^ provides an overview of the mechanisms available to submitters to request feedback from or a meeting with the Food and Drug Administration (FDA). The FDA supports medical device innovators.^82^ The FDA initiated a Software Precertification (Pre-Cert) Pilot Program as a future regulatory model to provide “more streamlined and efficient regulatory oversight of software-based medical devices developed by manufacturers who have demonstrated a robust culture of quality and organizational excellence and who are committed to monitoring the real-world performance of their products once they reach the U.S. market.” … “The FDA is partnering with a Federally Funded Research and Development Center (FFRDC), operated by The MITRE Corporation (MITRE), to provide professional engineering and technical support to simulate scenarios for the Software Pre-Cert Pilot Program,” ….’’The simulated scenarios will allow the agency to test the interdependencies of the four components of the Pre-Cert Pilot Program (Excellence Appraisal, Review Pathway Determination, Streamlined Premarket Review Process, and Real-World Performance).^83^ “

There may still be no specific legally binding regulation for MLMD, even in the United States. But the FDA is developing concepts and has been engaged in research and communication with stakeholders for several years.^84^ Regulation of AI intelligence in medical imaging considers three criteria: patient safety or device risk class, existence of a predicate algorithm or evolutionary/revolutionary devices, and clinician input (decision support, computer-aided detection, and computer-aided diagnosis) (56).

The Food and Drug Administration (FDA) proposed an advanced regulatory concept in the discussion paper, ‘‘Proposed Regulatory Framework for Modifications to Artificial Intelligence/Machine Learning (AI/ML)-Based Software as a Medical Device (SaMD).’’^85^ Two-dimensional risk classification is adopted from IMRDF.^86^ Changes in AI software are classified as changes in performance, input, or intended purpose. The total product lifecycle regulatory approach (TPLC) as specified in the Pre-Cert-Program is applied to the assessment of modifications. Good machine learning practices (GMLP)^87^ are supposed to assure process quality in data management, development, model training and tuning, model validation, and monitoring. Based on a premarket assessment of safety, performance, and clinical benefit, the FDA may permit the model to be marketed conditional on monitoring. The manufacturer is expected to pre-specify expected changes (SPS) due to retraining and retuning. Moreover, he has to provide an algorithm change protocol (ACP) in advance, where methods for controlling anticipated risks related to SPS are established.

The FDA published an update of its policy, the ‘‘Artificial Intelligence/Machine Learning (AI/ML)-Based Software as a Medical Device (SaMD) Action Plan.’’^88^ The FDA strongly advocates a multistakeholder approach with a particular focus on the needs of patients and takes part in regulatory research and harmonization of technical standards. Currently the FDA will check additional state-of-art requirements according to ‘‘General Features of Medical Device Regulation’’ section 10. For high-risk devices outcome data on safety, performance, and equity may be requested. Testing performance of MLMD algorithms across different imaging devices, and different provider and patient settings demands appropriate data sets. Certify-AI is a service of the Data Science Institute of the American College of Radiology that allows developers of health care AI to test independently and compliant with regulatory expectations the algorithm performance ahead of any regulatory review by the FDA or other governmental agencies.^89^ Other useful services are provided by this institute.^90^ A regulatory reference lab for testing MLMD with undisclosed representative big data would provide comparability, standardization and a minimum level of generalization. Ongoing monitoring and updating of the data would be required, however.

#### Other Legislations

In the United Kingdom, medical devices are regulated by the Medical Devices (Amendment, etc.) (EU Exit) Regulations 2020.^91^ The Medicines and Healthcare products Regulatory Agency (MHRA) is responsible for United Kingdom medical devices. CE-marked devices will be accepted until 30 June 2023. A United Kingdom-approved body must be used in cases where third-party conformity assessment is required for the revived UKCA marking. UKNI marking, conforming with EU and UK legislation will be necessary for the future of Northern Ireland.^92^ Brexit opportunities provide an outlook for some independent adaptation of the regulation of software and artificial intelligence as a medical device.^93^ ‘‘Software and AI as a Medical Device Change Programme’’^94^ is an initiative to develop UK regulatory guidance by the Medicines and Healthcare products Regulatory Agency (MHRA).^95^

The promises and challenges of MLMD are global issues entailing modernization of regulation everywhere, e.g., in Japan (57), China,^96^ Korea,^97^ and Saudi Arabia.^98^

#### Comparison of Eu and United States With Examples

**FIGURE 1:**
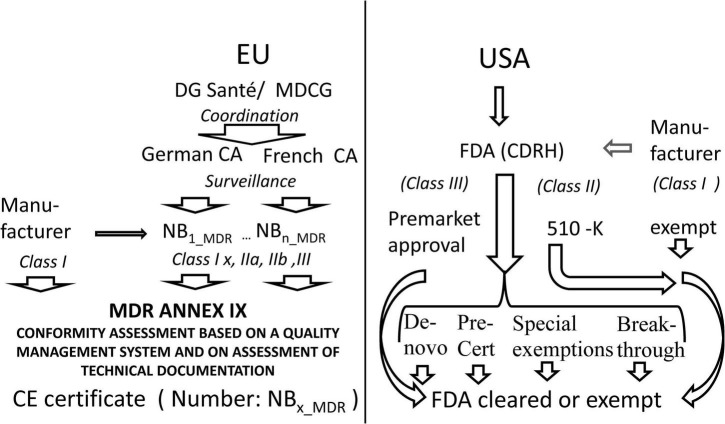
Comparison of access to the market in Europe as compared to the United States. MDCG Medical Device Coordination Group, CA Competent Authority, NB Notified Body, MDR Medical Device Regulation, CE certificates issued by a notified body bear a number identifying the notified body, FDA US Food and Drug Administration, CDRH FDA Center for Devices and Radiological Health.

The current regulatory pathway in the EU and in the United States is sketched in Figure 1.

Whereas, the United States maintains a centralized regulation with many tailored ways of access to the market the EU has reformed its decentralized CE-certification approach implementing stricter direct law in EU, improved oversight by competent authorities, and better coordination by the Commission.

In the past between 2015 and 2020 240 MLMD were CE marked in Europe and 222 were approved by the FDA. Twenty seven percent of all 462 MLMD were marketed in the EU and in the United States. The 510(k) pathway was used for 92% of FDA approvals, 7% used the *de novo* pathway and 1% were marketed by the premarket approval pathway (58).^99^

The problem is that this comparison is history. In the past, European regulation was based on the Council Directive 93/42/EEC and was more permissive than FDA. Now the recently implemented MDR is supposed to be a much stricter legislation in Europe, whereas the FDA has embraced the regulation of new technologies. The United Kingdom pathway is currently evolving from the European pathway and addresses a lot of technical issues related to transition periods and the particular requirements for Northern Ireland. Regulation of AI is evolving all over the world. Thus, what was approved in the past may differ from what will be approved in the future. In view of the current deployment of a new medical device regulation in Europe and the United Kingdom the impact on marketing MLMD devices cannot be compared systematically at the moment.

ChestLink is the first Class IIb MDR certified automation of radiological imaging workflow by MLMD without any involvement from a radiologist in Europe.^100^ “Prior to certification, ChestLink has been operating in a supervised reporting setting in multiple pilot locations for more than a year, processing more than 500,000 real-world chest X-ray images. Prior to autonomous operations, ChestLink deployments start with a retrospective imaging audit. Retrospective analysis helps to identify what part of studies at the medical institution can be successfully automated. The operations then move into a supervised setting, where ChestLink reports are validated by the Oxipit medical staff and radiologists at the medical institution. Only after completing the initial stages, the application can start to report autonomously. Fully autonomous ChestLink operations in a clinical setting are expected to begin in early 2023’’^101^ !

MLMD devices cleared by the FDA are listed along with further links on a special FDA website.^102^ Moreover, the data science institute of the American College of Radiology provides detailed and searchable information regarding FDA cleared AI medical imaging products.^103^ Some examples were also presented at the ‘‘Public Workshop--Evolving Role of Artificial Intelligence in Radiological Imaging. 25--26 February 2020’’ organized by the FDA.^104^

In 2020, the FDA authorized marketing of the first cardiac ultrasound software that guides users by artificial intelligence^105^ by 510(k) premarket notification. The Caption Guidance software is intended to assist medical professionals in the acquisition of cardiac ultrasound images. The Caption Guidance software is an accessory to compatible general purpose diagnostic ultrasound systems and classified as class II device. A prospective, multicenter diagnostic showed that novices without experience in ultrasonography using this device may obtain diagnostic transthoracic echocardiographic studies (59). So far, most of the devices approved by the FDA are designed to augment--but not entirely automate--the process of reviewing images and making diagnoses. IDx-DR is marketed as the first and only FDA cleared AI diagnostic system to make a diagnosis without physician input. IDX-DR is an autonomous AI-enabled device that detects more than mild diabetic retinopathy.^106^ Regulatory pathway was a 510(k) Premarket Notification with the predecessor as predicate device.^107^ This previous version was *De Novo*-cleared by the FDA 2018 as ophthalmic retinal diagnostic software device (class II).^108^ ‘‘IDx-DR is indicated *for use by healthcare providers* (italics by the author) to automatically detect more than mild diabetic retinopathy in adults diagnosed with diabetes who have not been previously diagnosed with diabetic retinopathy.^109^ “A recent meta-analysis of studies found that state-of-the-art, ML-based DR screening algorithms are ready for clinical applications. However, a significant portion of the earlier studies had methodology flaws and should be interpreted with caution (60).

There are two issues with IDx-DR. First it is a screening application, which may be used by non-specialist providers. Complex interactions between algorithmic output and providers may occur. Automation bias and the impact on medical-legal risk and responsibility have to be addressed. MLMD screening tools should be evaluated in large population-based, real-world settings with longitudinal data collection and linkage to regional registries. Last not least regulation needs to be adapted to exploit the benefits of continuously improving MLMD (61).

The second issue is that IDx-DR is an MLMD solution that automates a process in medical imaging on the most advanced autonomy level (full automation) (62). A binary classification of algorithms into assisting and autonomous ones does not adequately capture the spectrum of MLMD autonomy. Five levels of automation have been established in the automotive industry, full automation being the most advanced and most rare level.^110^ A model of graded autonomy should be based on central determinants of autonomy-related risk. The latter comprise the responsible agent monitoring and responding to medical events, the availability of a bail out back-up decision solution, and the specific context,^111^ where the algorithm is deployed. As the real-world performance of MLMD in previously untested clinical scenarios is unknown, any, in particular autonomous, MLMD systems require closely supervised pilot testing and controlling in real-world clinical settings (62) (->example ChestLink). Autonomous models in medical imaging should use multi−site heterogeneous data sets to ensure a minimum level of generalizability across diverse patient populations as well as variable imaging equipment and imaging protocols. Additional post−market surveillance is to ensure that algorithms function as expected longitudinally.^112^ Autonomous MLMD should be evaluated based on outcomes regarding safety, performance and equity in preregistered prospective studies, not only by comparison to standard of care (62).

Automation by MLMD might affect providers in unexpected ways (62). For example, skills rarely practiced may be lost, or training may change performance in the health-care providing human-agent-team. Risk management has to consider early warning systems and mitigation measures. The difficulty in guessing unexpected behavior of autonomous systems differs between deterministic models and black-box models. eXplainable artificial intelligence is of limited value in this high-stake context as interpretability (see section “Specific Risks by MLMD-Ethical and Legal Concerns” last paragraph) is not reliable in decision making for individual patients (63). For example, -a conventional thresholding algorithm designed to automatically segment 4d flow images of big vessels may be based on a model derived from data. The model is represented by deterministic code in the medical device software. Limitations of this model representing a potential hazard may be accounted for in the risk management process. Whereas in case of an autonomous MLMD model, -even if it is based on the same data-, predictions of unexpected behavior and corrective and preventive measures are much more difficult due to a lack of mechanistic understanding in individual cases. Moreover, advanced automation of MLMD is raising a serious liability issue in case of medical errors (62).

Thus, the FDA distinguishes between autonomous radiology AI as ‘‘software in which AI/ML is being used to automate some portion of the radiological imaging workflow’’ and ‘‘augmented intelligence’’ innovations currently on the market. Collateral change or challenge to the standard of care and introduction of new questions of safety and effectiveness into an established radiological imaging workflow or even new intended use including user and/or environment are considered as potential side effects of automation of radiological imaging workflow by MLMD.^113^ The American College of Radiology and the RSNA made a comment on this issue discussed at the “Public Workshop − Evolving Role of Artificial Intelligence in Radiological Imaging’’ of the FDA. The professional associations expressed concerns regarding autonomous AI lacking oversight by expert physicians. Combining expert professional judgment with MLMD algorithm is considered safer and more effective than unsupervised automation. It is contested that IDx-DR is an apt example demonstrating how autonomous AI could work in medical imaging, as the output does not recommend treatment but ophthalmologic referral. In radiology the output may pose a much greater risk for patient safety.^114^

## Summary and Reflections on the Future of Regulation

In summary, cardiac imaging will be increasingly pervaded by MLMD. It is a win-win combination (8) expected to enhance medical research, diagnosis, and treatment’s overall quality and efficiency and decrease radiation by sparse image reconstruction techniques (64). Automation of time-consuming human tasks, e.g., in image segmentation and screening, and improvements in the efficiency of imaging pipelines should result in a cost reduction.

Artificial intelligence and machine learning systems, e.g., the software controlling radiology devices, may directly impact real-world human-agent teams. Concerns regard ethics, law, including liability, data protection, medical device regulation, and the robustness of applications in various partly unforeseeable use scenarios. Problem areas are data privacy, enhanced vulnerability to cyber-attacks, bias fed into the model by prejudices deeply hidden in real-world training data or by non-representative or inappropriate sampling, lack of transparency and interpretability of the mapping of input to output, the complexity of software, generalizability of applications in various socio-technical ecosystems, potentially uncontrolled autonomous behavior of technical systems, and liability issues arising if an adverse event happens. Liability issues are discussed in a commission report of the European Union, where a risk-based revision of product liability legislation is recommended (65). Current regulation is based on regulation for software as a medical device. Additional legal constraints are data protection law and emerging artificial intelligence legislation.

Analysis of gaps in the current regulation of artificial intelligence-based diagnostic imaging algorithms in Europe and the United States yielded conflation of task and algorithm, superficial treatment of diagnostic task definition, lack of comparability, insufficient characterization of safety and performance elements, lack of resources to validate performance at every installation site, and inherent conflicts of interest as issues (52). The authors suggested that a definition of a task should include background information, a precise and detailed description, elaborated labeling instructions and prototypical examples, and counter-examples. Tasks should be separated from algorithms and maintained by medical societies or third parties with domain expertise. Performance elements beyond accuracy should be specified as reliability, applicability, deterministic non-distractible behavior, self-awareness of limitations, fail-safe function, transparent logic, confidence, ability to be monitored and audited, and usability. To manage limited resources for validation development of a simplified approach to validate and monitor on-site installation, e.g., out-of-distribution screening (66) is recommended (52).

Legislations worldwide are currently trying to adapt their regulation in an innovation-friendly manner to reap the promises of MLMD. There are two views on regulation, *ex ante* and *ex post*. The *ex ante* precautionary principle approach anticipates risks and imposes limits or bans on application development. The permissionless innovation approach allows experimentation and addresses emerging issues *ex post*. Both approaches compete with time in the rapidly evolving technology field (53). The goal to foster innovation favors the permissionless innovation approach. However, due to ethical and legal concerns, anticipation and mitigation of *ex ante* risks cannot be dismissed. Moreover, continuous monitoring, a fast adaptation of regulation, and resilient management of emerging issues will decide on a successful balance between innovation and regulation. Last but not least, the concept of risk and safety is at stake. Risk in the regulation of medical devices is generally conceived as a combination of the probability of occurrence and the severity of an adverse event. This negative concept must be differentiated from positive risk implying opportunity. The scope of this broader concept of risk is making decisions under uncertainty and supporting risk management.^115^ Certain risks are inherent in intended use and performance. Though a ship may be most ‘‘safe’’ in the harbor, the use implies the risk of going to sea. Safety in the future will be delivered in an integrated system in which humans, machines, and the environment work together and demand a more proactive view on risks.^116^

Whereas conventional approaches to technology regulation focus on manufacturing, MLMD regulations have to consider providers and patients applying MLMD in increasingly complex healthcare environments. Assuring the quality of structure and processes may go beyond the excellence of manufacturers. Applying MLMD must be part of professional medical discretion and patient education. Suppose MLMD applications are not frozen at the entry into the market. In that case, continuous learning will be associated with drifts and shifts in system behavior that needs surveillance and may require corrective and preventive actions. Thus, a total product lifecycle approach (TLPC) and multi-stakeholder involvement are essential features of any ethical or legal approach to environments comprising MLMD agents and humans and any technical risk-management framework concerning MLMD. Regulatory outlets supporting continuous learning are an issue in Europe (67).

**FIGURE 2:**
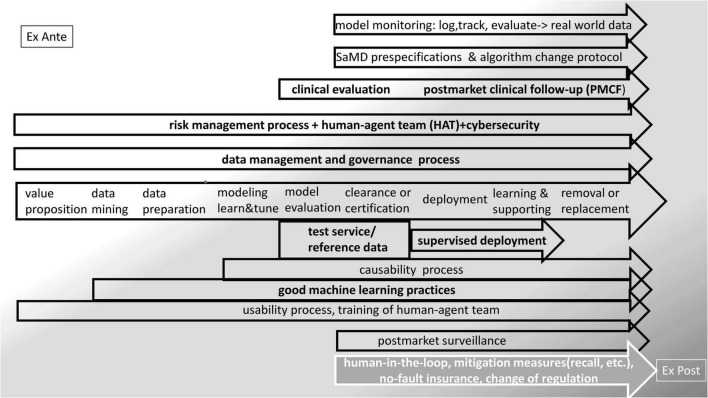
Embedding the MLMD life-cycle in regulation—existing approaches and options.

Some existing approaches and options to embed the MLMD lifecycle in a world of processes that provides control and adaptive learning is presented in Figure 2. MLMD is data greedy. Real-world data (RWD) is data derived from real-life patient management. Processes associated with health care provision leave digital traces that accumulate in storage systems and networks. Digital health generates big data. RWD comprises registries, electronic health records, picture archives, insurance data, health apps, digital traces in the internet of things (IoT), and social media. New data-mining techniques, for instance, a combination of natural language processing, NLP), machine learning, and robotic process automation, enable the analysis of these sources of information. Real-world evidence (RWE) is the information extracted from RWD (68–70). Thus, data governance in MLMD is a fundamental issue. A data-centric life cycle approach to MLMD is advocated for ethical (71) and technical reasons (72) and may be particularly helpful in health RWD that generally lacks maintenance and validation and raises many interoperability problems.

Regulatory science evaluates and develops the regulatory landscape and its environment. Advancing regulatory science is a crucial matter of concern for the FDA.^117^ The FDA has a strategic plan to support regulatory science and cooperates with academic science in the Centers of Excellence in Regulatory Science and Innovation (CERSIs) on this goal.^118^ The FDA maintains a science laboratory, the Office of Science and Engineering Laboratories (OSEL).^119^ ‘‘Artificial Intelligence and Machine Learning Program: Research on AI/ML-Based Medical Devices’’ is an OSEL project.^120^ Regulatory science is an issue worldwide promoted by dedicated organizations,^121^ CERSIs, and other universities.^122^ Regulatory science initially emerged in the pharma domain (73, 74). Regulatory science concerning medical devices is a recent achievement expected to help modernize regulation (75–77).

## Conclusion

The emerging disruption in the socio-technical environment by MLMD devices is driving the modernization of regulation. This also applies to health management, where particular ethical assets are at stake. A data-centric total product life-cycle approach, including all stakeholders, continuous learning of all agents in the human agent team, fast adaptation of regulation, and new, more proactive risk management may be salient ingredients of regulatory control of MLMD devices.

## Author Contributions

EW conceived the manuscript, compiled and reviewed relevant sources, and wrote the article.

## Conflict of Interest

The author declares that the research was conducted in the absence of any commercial or financial relationships that could be construed as a potential conflict of interest.

## Publisher’s Note

All claims expressed in this article are solely those of the authors and do not necessarily represent those of their affiliated organizations, or those of the publisher, the editors and the reviewers. Any product that may be evaluated in this article, or claim that may be made by its manufacturer, is not guaranteed or endorsed by the publisher.

## Footnotes

1 https://www.deeplearningbook.org accessed 27.4.2022.

2 https://www.image-net.org/challenges/LSVRC/ and https://cocodataset.org/#home and http://host.robots.ox.ac.uk/pascal/VOC/ accessed 27.4.2022.

3 https://caffe.berkeleyvision.org/ accessed 26.4.22.

4 https://www.tensorflow.org/ accessed 26.4.22.

5 https://pypi.org/project/Theano/ accessed 26.4.22.

6 https://pytorch.org/ accessed 26.4.22.

7 https://lasagne.readthedocs.io/en/latest/ accessed 26.4.22.

8 https://keras.io/ accessed 21.6.22.

9 FDA-cleared AI devices lack critical information on performance, equity. https://medcitynews.com/2021/04/fda-cleared-ai-devices-lack-critical-information-on-performance-equity/?rf=1 accessed 10.1.22.

10 https://www.imdrf.org/sites/default/files/2022-05/IMDRF%20AIMD%20WG%20Final%20Document%20N67.pdf (accessed June 21, 2022).

11 FDA Authorizes Marketing of First Cardiac Ultrasound Software That Uses Artificial Intelligence to Guide User. https://www.fda.gov/news-events/press-announcements/fda-authorizes-marketing-first-cardiac-ultrasound-software-uses-artificial-intelligence-guide-user accessed 12.1.22.

12 Radiological Society of North America (RSNA) Quantitative Imaging Biomarkers Alliance (QIBA) https://www.rsna.org/research/quantitative-imaging-biomarkers-alliance accessed 12.1.22.

13 European Society of Radiology (ESR) European Imaging Biomarkers Alliance (EIBALL) https://www.myesr.org/research/european-imaging-biomarkers-alliance-eiball accessed 12.1.22.

14 This algorithm may be adapted to cardiovascular imaging as it is open source under the GNU General Public License on GitHub https://github.com/ArnaoutLabUCSF/cardioML/tree/master/JACC_2021 accessed 12.1.22.

15 https://wellcome.org/sites/default/files/ai-in-health-ethical-social-political-challenges.pdf accessed 11.02.2022.

16 Article 4 (4) and Article 22 and Recitals (70) and (71) of the GDPR. https://eur-lex.europa.eu/legal-content/EN/TXT/HTML/?uri=CELEX:32016R0679&from=EN#d1e2793-1-1 accessed 12.02.2022.

17 https://digital-strategy.ec.europa.eu/en/policies/expert-group-ai accessed 11.02.2022.

18 “*Audience and Scope* These guidelines are addressed to all AI stakeholders designing, developing, deploying, implementing, using or being affected by AI, including but not limited to companies, organizations, researchers, public services, government agencies, institutions, civil society organizations, individuals, workers and consumers.” https://ec.europa.eu/futurium/en/ai-alliance-consultation.1.html accessed 27.04.2022.

19 https://digital-strategy.ec.europa.eu/en/library/assessment-list-trustworthy-artificial-intelligence-altai-self-assessment accessed 12.2.2022.

20 Explicability as an ethical principle of trustworthy AI comprises accountable use and is not identical to explainability in the technical context of XAI 45. (78).

21 The quality of use and usability are essential requirements for medical devices.

22 See discussion on autonomous devices.

23 https://www.imdrf.org/ accessed 14.2.21.

24 https://www.imdrf.org/sites/default/files/docs/imdrf/final/procedural/imdrf-proc-n39-strategic-plan-200925.pdf accessed 14.2.21.

25 The GHTF was replaced by the International Medical Device Regulators Forum (IMDRF) in 2011. GHTF Documents are available on the IMDRF web-site.

26 https://www.imdrf.org/sites/default/files/docs/ghtf/final/sg1/technical-docs/ghtf-sg1-n15-2006-guidance-classification-060627.pdf accessed 27.4.2022.

27 *“active device’ means any device, the operation of which depends on a source of energy other than that generated by the human body for that purpose, or by gravity, and which acts by changing the density of or converting that energy. Devices intended to transmit energy, substances or other elements between an active device and the patient, without any significant change, shall not be deemed to be active devices. Software shall also be deemed to be an active device;”* MDR art. 2 definition (4).

28 https://www.imdrf.org/search?search=SaMD&facets_query=&f%5B0%5D=content_type%3Atechnical_document accessed 14.2.21.

29 https://www.imdrf.org/sites/default/files/docs/imdrf/final/technical/imdrf-tech-140918-samd-framework-risk-categorization-141013.pdf accessed 27.4.2022.

30 https://eur-lex.europa.eu/legal-content/EN/TXT/PDF/?uri=OJ:L:2017:117:FULL&from=EN accessed 22.2.2022.

31 https://www.fda.gov/media/114034/download accessed 27.4.2022.

32 https://www.fda.gov/media/85284/download accessed 27.4.2022.

33 https://eur-lex.europa.eu/resource.html?uri=cellar:e0649735-a372-11eb-9585-01aa75ed71a1.0001.02/DOC_1&format=PDF and https://eur-lex.europa.eu/resource.html?uri~=~cellar:e0649735-a372-11eb-9585-01aa75ed71a1.0001.02/DOC_2&format~=~PDF accessed 22.2.2022.

34 https://www.fda.gov/media/122535/download accessed 28. 2.2022.

35 https://www.fda.gov/media/71794/download accessed 27.4.2022.

36 https://docs.pytest.org/en/7.1.x/ accessed 27.4.2022.

37 https://csrc.nist.gov/projects/automated-combinatorial-testing-for-software accessed 27.4.2022.

38 https://paperswithcode.com/ accessed 27.4.2022.

39 https://www.cdc.gov/phlp/publications/topic/hipaa.html accessed 14.2.2022.

40 https://www.cencenelec.eu/ accessed 27.4.2022.

41 https://ansi.org/ accessed 27.4.2022.

42 https://www.nist.gov/ accessed 27.4.2022.

43 https://www.bsigroup.com/ accessed 27.4.2022.

44 https://www.iso.org/home.html accessed 27.4.2022.

45 https://www.ieee.org/ accessed 27.4.2022.

46 https://www.iso.org/standard/59752.html accessed 14.2.2022.

47 https://www.iso.org/standard/54534.html accessed 14.2.2022.

48 https://www.iso.org/standard/59543.html accessed 14.2.2022.

49 Covers SaMD and health software that is no medical device. The new version was not accepted.

50 https://www.iso.org/standard/78215.html accessed 14.2.2022.

51 https://www.iso.org/standard/72704.html accessed 14.2.2022.

52 https://www.iso.org/standard/63179.html accessed 14.2.2022.

53 https://www.iso.org/standard/71690.html accessed 14.2.2022.

54 https://www.iso.org/standard/38421.html accessed 14.2.2022.

55 https://www.iso.org/standard/76097.html accessed 14.2.2022.

56 https://www.itu.int/en/ITU-T/focusgroups/ai4h/Pages/default.aspx accessed 14.2.2022.

57 https://www.itu.int/en/ITU-T/focusgroups/ai4h/Documents/FG-AI4H_Whitepaper.pdf accessed 22.2.2022.

58 https://www.bsigroup.com/globalassets/localfiles/en-gb/about-bsi/nsb/innovation/mhra-ai-paper-2019.pdf accessed 14.2.2022.

59 https://webstore.iec.ch/publication/67138 accessed 14.2.2022.

60 https://standards.ieee.org/wp-content/uploads/import/documents/other/ead_v2.pdf accessed 28.2.2022.

61 https://www.accessdata.fda.gov/scripts/cdrh/cfdocs/cfStandards/search.cfm accessed 28.4.2022.

62 Harmonized Standards (HAS)-consultants.

63 Article 8 MDR/IVDR.

64 https://eur-lex.europa.eu/legal-content/EN/TXT/PDF/?uri=CELEX:32022D0006&from=EN accessed 28.4.2022.

65 https://eur-lex.europa.eu/legal-content/EN/TXT/PDF/?uri=OJ:L:2017:117:FULL&from=EN accessed 22.2.2022.

66 https://eur-lex.europa.eu/legal-content/EN/TXT/PDF/?uri=OJ:L:2016:119:FULL&from=EN accessed 22.2.2022.

67 https://digital-strategy.ec.europa.eu/en/policies/regulatory-framework-ai accessed 22.2.2022.

68 https://eur-lex.europa.eu/resource.html?uri=cellar:e0649735-a372-11eb-9585-01aa75ed71a1.0001.02/DOC_1&format=PDF and https://eur-lex.europa.eu/resource.html?uri~=~cellar:e0649735-a372-11eb-9585-01aa75ed71a1.0001.02/DOC_2&format~=~PDF accessed 22.2.2022.

69 https://portolano.it/en/blog/life-sciences/artificial-intelligence-and-medical-devices-the-potential-impact-of-the-proposed-artificial-intelligence-regulation-on-medical-device-software accessed 27.4.2022.

70
*Rule 11 of Annex VIII MDR. “Software intended to provide information which is used to take decisions with diagnosis or therapeutic purposes is classified as class IIa, except if such decisions have an impact that may cause: Death or an irreversible deterioration of a person’s state of health, in which case it is in class III; or Serious deterioration of a person’s state of health or a surgical intervention, in which case it is classified as class IIb. Software intended to monitor physiological processes is classified as class IIa, except if it is intended for monitoring of vital physiological parameters, where the nature of variations of those parameters is such that it could result in immediate danger to the patient, in which case it is classified as class IIb. All other software are classified as class I.” Rule 22 of Annex VIII MDR: “Active therapeutic devices with an integrated or incorporated diagnostic function which significantly determines the patient management by the device, such as closed loop systems or automated external defibrillators Class III.”*

71 https://ec.europa.eu/health/system/files/2020-09/md_mdcg_2019_11_guidance_qualification_classification_software_en_0.pdf accessed 27.4.2022.

72 https://www.imdrf.org/sites/default/files/docs/imdrf/final/technical/imdrf-tech-140918-samd-framework-risk-categorization-141013.pdf accessed 27.4.2022.

73 https://digital-strategy.ec.europa.eu/en/library/impact-assessment-regulation-artificial-intelligence accessed 22.2.2022.

74 https://ec.europa.eu/growth/tools-databases/nando/index.cfm?fuseaction=directive.notifiedbody&dir_id=34 accessed 22.2.2022.

75 https://ec.europa.eu/tools/eudamed/#/screen/home accessed 22.2.2022.

76 https://ec.europa.eu/health/system/files/2022-01/md_cybersecurity_en.pdf accessed 22.2.2022.

77 https://github.com/johner-institut/ai-guideline accessed 1.5.2022.

78 https://www.accessdata.fda.gov/scripts/cdrh/cfdocs/cfcfr/CFRSearch.cfm?CFRPartFrom=800&CPartTo=1299 accessed 28. 2.2022.

79 https://www.fda.gov/medical-devices/device-advice-comprehensive-regulatory-assistance/overview-device-regulation accessed 28. 2.2022.

80 https://www.fda.gov/regulatory-information/search-fda-guidance-documents/breakthrough-devices-program accessed 1.5.2022.

81 https://www.fda.gov/media/114034/download accessed 1.5.2022.

82 https://www.fda.gov/about-fda/cdrh-innovation/activities-support-medical-device-innovators#earlyworkshop accessed 1.5.2022.

83 https://www.fda.gov/medical-devices/digital-health-center-excellence/digital-health-software-precertification-pre-cert-program accessed 28. 2.202.

84 https://www.fda.gov/medical-devices/software-medical-device-samd/artificial-intelligence-and-machine-learning-software-medical-device accessed 1.5.2022.

85 https://www.fda.gov/media/122535/download accessed 1.5.2022.

86 https://www.imdrf.org/sites/default/files/docs/imdrf/final/technical/imdrf-tech-140918-samd-framework-risk-categorization-141013.pdf accessed 1.5.2022.

87 https://www.fda.gov/medical-devices/software-medical-device-samd/good-machine-learning-practice-medical-device-development-guiding-principles?utm_medium=email&utm_source=govdelivery accessed 28. 2.2022.

88 https://www.fda.gov/media/145022/download accessed 1.5.2022.

89 https://www.acrdsi.org/DSI-Services/Certify-AI accessed 1.5.2022.

90 https://www.acrdsi.org/DSI-Services accessed 1.5.2022.

91 https://www.legislation.gov.uk/uksi/2020/1478/contents/made accessed 28. 2.2022.

92 https://www.gov.uk/guidance/regulating-medical-devices-in-the-uk accessed 28. 2.2022.

93 https://www.gov.uk/government/news/transforming-the-regulation-of-software-and-artificial-intelligence-as-a-medical-device accessed 28. 2.2022.

94 https://www.gov.uk/government/publications/software-and-ai-as-a-medical-device-change-programme/software-and-ai-as-a-medical-device-change-programme accessed 28. 2.2022.

95 https://www.gov.uk/government/organisations/medicines-and-healthcare-products-regulatory-agency accessed 28. 2.2022.

96 https://chinameddevice.com/china-cfda-ai-software-guideline/ accessed 28. 2.2022.

97 https://www.mfds.go.kr/eng/brd/m_40/view.do?seq=72623&srchFr=&srchTo=&srchWord=&srchTp=&itm_seq_1=0&itm_seq_2=0&multi_itm_seq=0&company_cd=&company_nm=&page=1 accessed 28.2.2022.

98 https://sfda.gov.sa/index.php/en/regulations/79702 accessed 28. 2.2022.

99 https://www.thelancet.com/cms/10.1016/S2589-7500(20)30292-2/attachment/c8457399-f5ce-4a30-8d36-2a9c835fb86d/mmc1.pdf accessed 1.5.2022.

100 https://grand-challenge.org/aiforradiology/product/oxipit-chestlink/ accessed 1.5.2022.

101 https://www.pr.com/press-release/858165 accessed 1.5.2022.

102 https://www.fda.gov/medical-devices/software-medical-device-samd/artificial-intelligence-and-machine-learning-aiml-enabled-medical-devices accessed 1.5.2022.

103 https://aicentral.acrdsi.org/ accessed 1.5.2022.

104 https://www.fda.gov/medical-devices/workshops-conferences-medical-devices/public-workshop-evolving-role-artificial-intelligence-radiological-imaging-02252020-02262020 accessed 1.5.2022.

105 https://aicentral.acrdsi.org/ProductDetails/173 accessed 1.5.2022.

106 https://www.digitaldiagnostics.com/products/eye-disease/idx-dr/ accessed 1.5.2022.

107 https://www.accessdata.fda.gov/scripts/cdrh/cfdocs/cfPMN/pmn.cfm?ID=K203629 accessed 1.5.2022.

108 https://www.accessdata.fda.gov/cdrh_docs/reviews/DEN180001.pdf accessed 1.5.2022.

109 https://www.accessdata.fda.gov/cdrh_docs/pdf20/K203629.pdf accessed 1.5.2022.

110 https://www.sae.org/standards/content/j3016_202104 accessed 1.5.2022.

111 medical domain, health-care system, and population…

112 https://www.acr.org/-/media/ACR/NOINDEX/Advocacy/acr_rsna_comments_fda-ai-evolvingrole-ws_6-30-2020.pdf accessed 1.5.2022.

113 https://www.fda.gov/media/135702/download Slide 7 accessed 1.5.2022.

114 https://www.acr.org/-/media/ACR/NOINDEX/Advocacy/acr_rsna_comments_fda-ai-evolvingrole-ws_6-30-2020.pdf accessed 1.5.2022.

115 IEC 31010:2019 Risk management—Risk assessment techniques https://webstore.iec.ch/publication/59809 accessed 28. 2.2022.

116 https://storage-iecwebsite-prd-iec-ch.s3.eu-west-1.amazonaws.com/2020-11/content/media/files/iec_wp_safety_in_the_future_en_lr_1.pdf accessed 28.2.2022.

117 https://www.fda.gov/science-research/science-and-research-special-topics/advancing-regulatory-science accessed 28. 2.2022.

118 https://www.fda.gov/science-research/advancing-regulatory-science/centers-excellence-regulatory-science-and-innovation-cersis accessed 28.2.2022.

119 https://www.fda.gov/medical-devices/science-and-research-medical-devices/medical-device-regulatory-science-research-programs-conducted-osel accessed 28.2.2022.

120 https://www.fda.gov/medical-devices/medical-device-regulatory-science-research-programs-conducted-osel/artificial-intelligence-and-machine-learning-program-research-aiml-based-medical-devices accessed 28.2.2022.

121 CIRS Centre for Innovation in Regulatory Science UK https://www.cirsci.org/, Institute for regulatory science https://nars.org/ accessed 28.2.2022.

122 Regulatory Affairs Office at Technical University Dresden https://digitalhealth.tu-dresden.de/infrastructure/regulatory-affairs-office/, Centre for Regulatory Science, A Newcastle University Centre of Research Excellence https://www.ncl.ac.uk/research/regulatory-science/ accessed 28.2.2022.
