# Supplementary material for: Real-World and Regulatory Perspectives of Artificial Intelligence in Cardiovascular Imaging
Source: Front Cardiovasc Med. 2022 Jul 22;9:890809. doi: 10.3389/fcvm.2022.890809 (PMC9354141; doi:10.3389/fcvm.2022.890809)
Supplement: Supplementary file 1 [file Data_Sheet_1.docx]

Supplementary Material

Appendix

Version 10.7.2022

Wellnhofer E (2022) Real-World and Regulatory Perspectives of Artificial Intelligence in Cardiovascular Imaging. Front. Cardiovasc. Med. 9:890809

Since the final submission and publication, some developments happened. There are some corrective amendments. Moreover, I would like to recommend some recent books for further reading.

1. **Recent developments**

IMDRF published a new technical document on the 9th of May

Machine Learning-enabled Medical Devices: Key Terms and Definitions

IMDRF Code IMDRF/AIMD WG/N67

[*https://www.imdrf.org/sites/default/files/2022-05/IMDRF%20AIMD%20WG%20Final%20Document%20N67.pdf*](https://www.imdrf.org/sites/default/files/2022-05/IMDRF%20AIMD%20WG%20Final%20Document%20N67.pdf) *accessed 21.6.2022*

IMDRF suggests that AI/ML medical devices be termed "Machine Learning Medical Devices (MLMD). I did replace “AI/ML medical devices” by “MLMD” in the paper except in direct quotes. **MLMD** is defined as:

*“A medical device that uses machine learning, in part or in whole, to achieve its intended medical purpose.”*

**Bias** is defined as:

*“Systematic difference in treatment 2 of certain objects, people, or groups in*

*comparison to others.*

*Note 1 to entry: Treatment is any kind of action, including perception, observation,*

*representation, prediction or decision. (ISO/IEC TR 24027:2021) Among the key definitions, the definition of bias suggests a citation and minor clarification/amendment.”*

IMDRF distinguishes wanted and unwanted bias depending on, whether bias supports the intended purpose of an AI(-based) system or not. In a screening application, a high sensitivity bias may be wanted. IMDRF bias definition is based on

ISO/IEC TR 24027:2021

Information technology — Artificial intelligence (AI) — Bias in AI systems and AI aided decision making.

<https://www.iso.org/standard/77607.html> *accessed 21.6.2022*

**Changes to MLMD**

IMDRF provides a definition of **continuous learning**:

*“Continuous Learning”* is *“Training that leads to change of an MLMD with each exposure to data that takes place on an ongoing basis during the operation phase of the MLMD life cycle. (Modified from ISO/IEC DIS 22989)*

*Note : Although not necessarily in opposition, Batch Learning is often referred to*

*when describing Continuous Learning. Batch Learning is a training that leads to*

*the change of an MLMD that involves discrete updates based on defined sets of*

*data that take place at distinct points prior to or during the operation phase of the*

*MLMD life cycle. “*

IMDRF discusses aspects that describe **changes to MLMD**.

These aspects “*describe what changes, as well as why, where, when, and*

*how the MLMD change occurs.”*

IMDRF introduces “**Changes to MLMD Environment for Data**”.

*“The cause of an MLMD environmental change refers to the source of the change*

*relative to the development environment. Examples of such causes include changes to the format or quality of the MLMD inputs (e.g., changes to third party image processing, incidents of adversarial machine learning); changes in the patient population (e.g., demographic shift); changes in clinical practice (e.g., earlier interventions that mask features used by the ML model for classification), etc.*

*The effect of an MLMD environmental change can involve deteriorated or improved*

*performance, effectiveness, or safety.*

*The domain of an MLMD environmental change refers to the scope or applicable extent of the change, which can be categorized as either homogeneous or heterogeneous. Heterogeneous changes are non-uniform changes that can be specific to one clinic, region, demographic, etc. (sometimes referred to as local changes). Homogeneous changes are changes that occur uniformly (universally, globally) over some groups or settings/context. Note that global does not denote around-the-world.”*

1. **Additional amendments:**

**Clarification & correction**

Not all machine learning approaches map data patterns on output without intermediate hypothesis modelling as suggested in the introduction of the paper (line 119). Some statistical and Bayesian models as well as rule-based approaches support explicit mechanistic modelling.

**Trends in Learning of MLMD**

Though currently continuously learning MLMD may not be certified, there are approaches to cover learning MLMD by regulation in Europe and in the USA.

**EU:**

“*For high-risk AI systems that continue to learn after being placed on the market or*

*put into service, changes to the high-risk AI system and its performance that have*

*been pre-determined by the provider at the moment of the initial conformity*

*assessment and are part of the information contained in the technical documentation*

*referred to in point 2(f) of Annex IV, shall not constitute a substantial modification.”*

Source: Second clause in Art. 43 paragraph 4 Artificial Intelligence Act (AIA)

<https://eur-lex.europa.eu/resource.html?uri=cellar:e0649735-a372-11eb-9585-01aa75ed71a1.0001.02/DOC_1&format=PDF> accessed 22.6.22

**USA:**

FDA expects “*manufacturers to monitor the AI/ML device and incorporate a risk management and other approaches outlined in “Deciding When to Submit a 510(k) for a Software Change to an Existing Device”^[[1]](#footnote-1)^ in development, validation, and execution of the algorithm changes (SaMD Pre-Specifications and Algorithm Change Protocol);…*

*The predetermined change control plan would include the types of anticipated modifications – SaMD Pre-Specifications – based on the retraining and model update strategy, and the associated methodology – Algorithm Change Protocol – being used to implement those changes in a controlled manner that manages risks to patients.”*

All changes of AI/ML based SaMD with approved SPS and ACP have to be documented. If the software modifications decision tree would require new 510(k) according to guidance “Deciding When to Submit a 510(k) for a Software Change to an Existing Device” and modifications are outside of agreed SPS+ACP but no new intended use is requested a focussed review is requested by FDA (new approved SPS+ACP). Modifications that lead to a new intended use require a FDA premarket review.

Source: “Proposed Regulatory Framework for Modifications to Artificial

Intelligence/Machine Learning (AI/ML)-Based Software as a Medical Device

(SaMD) - Discussion Paper and Request for Feedback”

<https://www.fda.gov/media/122535/download> accessed 22.6.22

**FDA strategy to foster excellence of manufacturer**

The FDA’s Center for Devices and Radiological Health (CDRH) issued a recent *“draft guidance to describe its policy regarding FDA’s participation in the Voluntary Improvement Program (VIP). The VIP is a voluntary program facilitated through the Medical Device Innovation Consortium (MDIC) that evaluates the capability and performance of a medical device manufacturer’s practices using third-party appraisals, and is intended to guide improvement to enhance the quality of devices.* *… The VIP builds on the framework piloted through FDA’s 2018 Case for Quality Voluntary Medical Device Manufacturing and Product Quality Pilot Program (CfQ Pilot Program) …This voluntary program is currently only available to eligible manufacturers of medical devices regulated by CDRH ….”*

*“As part of CDRH's 2016-2017 strategic priority to “Promote a Culture of Quality and Organizational Excellence,” CDRH envisions a future where the medical device ecosystem is inherently focused on device features and manufacturing practices that have the greatest impact on product quality and patient safety. Among its other regulatory activities, FDA evaluates manufacturers' compliance with regulations governing the design and production of devices. Compliance with the Quality System Regulation, 21 CFR Part 820, is a baseline requirement for medical device manufacturing firms. ….* *The practices are detailed in the Information Systems Audit and Control Association (ISACA) Capability Maturity Model Integration (CMMI) system.*

Source:

“Fostering Medical Device Improvement: FDA Activities and Engagement with the Voluntary Improvement Program. Draft Guidance for Industry and Food and Drug Administration Staff.”

<https://www.fda.gov/regulatory-information/search-fda-guidance-documents/fostering-medical-device-improvement-fda-activities-and-engagement-voluntary-improvement-program> accessed 22.6.22

The CDRH collaborates with ISACA training and certification organization as 3^rd^ party in the pilot program to assess and enhance organizational quality.

“*Beyond training and certification, ISACA’s CMMI models and platforms offer risk-focused programs for enterprise and product assessment and improvement”*

Source: <https://www.isaca.org/> accessed 22.6.22

**AI regulatory sandboxes**

*“AI regulatory sandboxes established by one or more Member States competent*

*authorities or the European Data Protection Supervisor shall provide a controlled*

*environment that facilitates the development, testing and validation of innovative AI*

*systems for a limited time before their placement on the market or putting into*

*service pursuant to a specific plan. This shall take place under the direct supervision*

*and guidance by the competent authorities with a view to ensuring compliance with*

*the requirements of this Regulation and, where relevant, other Union and Member*

*States legislation supervised within the sandbox.”*

Source: Art. 53 paragraph 1 Artificial Intelligence Act (AIA)

<https://eur-lex.europa.eu/resource.html?uri=cellar:e0649735-a372-11eb-9585-01aa75ed71a1.0001.02/DOC_1&format=PDF> accessed 22.6.22

Similar approaches are discussed in the USA (see paper).

**New EU-study**

June 2022, the thinktank of the European Parliament published a study:

**“Artificial intelligence in healthcare Applications, risks, and ethical and societal impacts”**

<https://www.europarl.europa.eu/thinktank/en/document/EPRS_STU(2022)729512>

<https://www.europarl.europa.eu/RegData/etudes/STUD/2022/729512/EPRS_STU(2022)729512_EN.pdf> accessed 9.7.2022

Seven main risk areas are identified:

1. Patient harm due to AI errors
2. Misuse of medical AI tools
3. Bias in AI
4. Lack of transparency
5. Privacy and security issues
6. Gaps in accountability (legal gaps in current regulations, difficulties in defining roles and responsibilities for multiple actors involved in medical AI, and lack of ethical and legal governance for AI manufacturers)
7. Obstacles in implementation

The study concludes that a more holistic, multi-faceted evaluation for future AI solutions in healthcare. Recommended policy options are:

1. Extension of AI regulatory frameworks and best practices to address healthcare-specific risks and requirements.
2. Promotion of multi-stakeholder engagement and co-creation throughout the MLMD lifecycle.
3. AI passport and traceability mechanisms that contain five categories of information:
   1. Model-related information
   2. Data-related information
   3. Evaluation -related Information
   4. Usage-related information
   5. Maintenance-related information
4. Addressing accountability and monitoring of responsibilities
5. Education and fostering of skills an literacy of the general public in medical AI
6. Promotion of further technical, clinical, ethical and regulatory research
7. Strategy for reducing the European divide in medical AI

Mitigation measures are recommended. Recent European consensus guidelines for trustworthy AI in medicine are based on EC-funded research and international inter-disciplinary experts.

FUTURE-AI: Best practices for trustworthy AI in medicine <http://future-ai.eu/> accessed 9.7.2022

**New FDA Guidance on quantitative imaging in radiology issued June 2022**

Technical Performance Assessment of Quantitative Imaging in Radiological Device Premarket

Submissions- Guidance for Industry and Food and Drug Administration Staff

<https://www.fda.gov/media/123271/download?utm_source=CleverReach&utm_medium=email&utm_campaign=05-05-22+Kampagnen-Name&utm_content=Mailing_13683449> accessed 9.7.2022

The guidance provides definitions and discusses sources of error, e. g. related to patient characteristics, image acquisition and processing. Information that must be provided for premarket submission is specified. A technical description of the quantitative imaging function(s) included in the device must provide sufficient detail for the Agency to understand the functionality. The report should include the level of automation (e.g., manual, automatic, or semi-automatic), and, if applicable, a brief summary of the algorithm training paradigm, including how algorithm parameters and thresholds were established. Moreover, information about input data (e.g., images) should include:

“*Restrictions on input data, such as imaging modalities, as applicable, (e.g., computed tomography, magnetic resonance), make, model, and specific trade name for each modality/system, specific ancillary hardware/software necessary to produce the input data (e.g., magnetic resonance elastography (MRE) acoustic driver), specific image acquisition parameter ranges (e.g., kVp range, slice thickness, voxel size) or specific imaging protocol(s) (e.g., pre-exam diet, breath hold, magnetic resonance angiography (MRA) sequence); or*

*Specific limitations, including diseases/conditions/abnormalities or imaging conditions, for which your quantitative imaging function has been found ineffective and should not be used, as applicable, Image acceptance activities (e.g., how your device ensures that input data (e.g., images)/preprocessing are acceptable for processing with your algorithm) and whether these are performed manually by a trained user or automatically by your algorithm,*

*Information presented to the user about the derived values (including units); and*

*The level of user interaction needed for your device to be used as intended, and if applicable, instructions for users (preprocessing image steps, selecting seed points, applying algorithm, and verifying resulting measurement for a lesion sizing tool)*”

Ten steps are recommended for technical performance assessment. These include the determination of an appropriate reference standard and applicable performance metrics. Bias, precision, limits of detection, limits of quantitation, linearity, sensitivity, specificity, and uncertainty should generally be considered. Acceptance criteria (performance targets or goals) should be chosen carefully based on the intended use of the quantitative imaging function and other restrictions/limitations (such as minimum image quality requirements).

The last section of recommendations concerns labelling.

The application of the guidance is illustrated in manual, semi-automatic and fully automatic examples.

**Remark on MLMD in the context of clinical decision making**

The following paper is a well written recent review identifying strengths, weaknesses, opportunities and threats of ML in a model of clinical decision making starting with data acquisition through the steps of feature extraction, interpretation, and decision-support:

Sanchez-Martinez S, Camara O, Piella G, Cikes M, González-Ballester MÁ, Miron M, et al. Machine Learning for Clinical Decision-Making: Challenges and Opportunities in Cardiovascular Imaging. Frontiers in cardiovascular medicine (2022) 8:765693-. doi: 10.3389/fcvm.2021.765693. PubMed PMID: 35059445.

I agree with Martinez et al. that the steps of data acquisition and feature extraction are most burdensome and tedious for humans, pose no immediate risk to patients, and should be automated. Interpretation may be enhanced by ML. Yet, though risk definitely becomes more immediate approaching medical decision, hidden errors or biases may corrupt evidence at earlier steps in a malicious way as demonstrated by Gichoya et al.:

*“The results from our study emphasise that the ability of AI deep learning models to predict self-reported race is itself not the issue of importance. However, our finding that AI can accurately predict self-reported race, even from corrupted, cropped, and noised medical images, often when clinical experts cannot, creates an enormous risk for all model deployments in medical imaging.”*

Gichoya JW, Banerjee I, Bhimireddy AR, Burns JL, Celi LA, Chen L-C, et al. AI recognition of patient race in medical imaging: a modelling study. The Lancet Digital Health. doi: 10.1016/S2589-7500(22)00063-2.

Thus, automation bias must be avoided by thorough analysis and evaluation of evidence prior to the decision. The vulnerabilities of only partially understood processes that are fed by data and generate evidence thereof need to be considered. Mitigation should include external validation if feasible.

1. **Recommendation of some recent books**

Artificial Intelligence in Cardiothoracic Imaging

Series Contemporary Medical Imaging.

Humana Cham (2022)

DOI: 10.1007/978-3-030-92087-6

<https://link.springer.com/book/10.1007/978-3-030-92087-6#bibliographic-information> accessed 29.6.2022

The Future of Medical Device Regulation: Innovation and Protection. Cambridge: Cambridge University Press (2022).

DOI: 10.1017/9781108975452

<https://www.cambridge.org/core/books/future-of-medical-device-regulation/7ABD9575718C5F31B69E4CE00DD7F7E7> accessed 29.6.2022

European Commission, Directorate-General for Health and Food Safety, Lupiáñez-Villanueva, F., Gunderson, L., Vitiello, S., et al., Study on health data, digital health and artificial intelligence in healthcare, Publications Office of the European Union, 2022, <https://data.europa.eu/doi/10.2875/702007> accessed 29.6.2022

1. <https://www.fda.gov/regulatory-information/search-fda-guidance-documents/deciding-when-submit-510k-software-change-existing-device> accessed 22.6.22. [↑](#footnote-ref-1)
